# Weightbearing versus non-weight bearing in geriatric distal femoral fractures: a systematic review and meta-analysis

**DOI:** 10.1007/s00068-024-02550-7

**Published:** 2024-05-22

**Authors:** Blaise Wardle, Joseph T. Lynch, Thomas Staniforth, Thomas Ward, Paul Smith

**Affiliations:** 1 Trauma and Orthopaedic Research Unit, Yamba Drive, Canberra, Australian Capital Territory 2605 Australia; 2 Canberra Hospital Orthopaedic Department, Canberra, Australian Capital Territory Australia; 3 The Australian National University, Canberra, Australian Capital Territory Australia

**Keywords:** Fragility fracture, Distal femur, Weight bearing, Complication, Non-union

## Abstract

**Background:**

Demographics of patients who sustain geriatric distal femoral fractures (DFF) match those of patients with neck-of-femur fractures but have limited evidence with which to support post-operative weightbearing protocols.

**Purpose:**

This systematic review sought to identify any difference in outcomes for elderly patients with DFF who were allowed early versus delayed weightbearing postoperatively.

**Methods:**

**Data sources:**

PubMed, Medline, Embase and The Cochrane Library, reference lists of retrieved articles.

**Study selection:**

English language papers published between January 2010 and February 2023 with AO-OTA type 33A, B and C femoral fractures as well as Lewis and Rorabeck Type I and II periprosthetic DFF surgically treated with either a lateral locking plate or retrograde intramedullary nail and an average patient age of ≥ 60 years.

**Data extraction:**

Studies were assessed for inclusion by two authors and quality was assessed using the MINORS tool.

**Data synthesis:**

Sixteen studies were included, Meta-analysis of non-union, malunion, infection, delayed union and implant complications was performed using Microsoft Excel and the MetaXL extension. The data on return to mobility were presented in narrative form. The analyses demonstrated no difference between the early and delayed weightbearing groups.

**Conclusions:**

There are no significant differences in complication rates between early versus delayed weightbearing after surgery for DFF in an elderly population. The study results are limited by high heterogeneity and low-quality studies. High quality, prospective studies are needed to determine the ideal postoperative weightbearing protocol.

**Level of evidence:**

Level III, Systematic Review and Meta-analysis of Level III studies.

International Prospective Register of Systematic Reviews registration—Prospero CRD42022371460.

**Supplementary Information:**

The online version contains supplementary material available at 10.1007/s00068-024-02550-7.

## Introduction

The management of geriatric distal femoral fractures (DFF) is a significant challenge in orthopaedic surgery. Controversy exists regarding the optimal timing for surgery, ideal constructs for fixation of these fractures and ideal postoperative weightbearing protocol [1, 2]. This is due to the relatively low incidence of these fractures, which makes it difficult to draw broad conclusions from single-centre studies.

DFF account for 3–6% of all femoral fractures [1, 3] and include periprosthetic fractures around total knee replacements, which have demonstrated an increased incidence with an ageing population [4]. These fractures are particularly challenging to manage in elderly patients due to decreased bone mineral density and represent a significant challenge for orthopaedic surgeons [1]. The demographics of geriatric patients who sustain DFF mirror those who sustain neck-of-femur fractures with multiple comorbidities, poor bone mineral density and often difficulty mobilising prior to the injury [5, 6]. As a result, there is a high morbidity and mortality from these injuries and a significant cost burden on the healthcare system [5].

Despite similarities to neck-of-femur fracture patients in age, mobility, cognitive status, and comorbidities [1, 7], treatment pathways which include multidisciplinary management, early surgical intervention, and early mobilisation have not been applied to DFF [1]. Postoperative weightbearing protocols constitute a spectrum from non-weightbearing (NWB) to fully weightbearing (FWB) or weightbearing as tolerated (WBAT). Some authors advocate for minimal restrictions and early weightbearing while others prefer to delay weightbearing until there is radiographic evidence of fracture healing [3].

Fear about failure of fixation and contradictory reports in the literature form a barrier to adopting a more aggressive approach to weightbearing for these patients. Excessive loading can lead to non-unions, but some loading can also be beneficial, further complicating the decision-making [8–10]. However, delaying weightbearing is not without risk. Studies have demonstrated the inability of geriatric patients to maintain weightbearing restrictions, meaning it may be an all-or-nothing proposition [11]. Delaying weightbearing presents a significant barrier in the mobilisation of geriatric patients with a greater burden on hospital staff as well as a higher risk of falls [9]. It can also contribute to thromboembolic events, pneumonia, pressure areas, infection, and exacerbates pre-existing sarcopenia which may preclude a return to pre-morbid mobility [12–15].

In hip fracture patients the effects of expeditious surgery and early mobilisation has been studied extensively [16]. Unfortunately, the same is not true for geriatric patients with DFF.

This study assesses the available literature relating to outcomes for patients with DFF based on postoperative weightbearing status. Comparing patients treated either with early weightbearing (EWB) or delayed weightbearing (DWB) in terms of morbidity, mortality, and functional outcomes.

## Methods

### Objectives

We focused on studies that evaluated the outcomes after surgical fixation of geriatric DFF and also documented the weightbearing protocol applied to these patients. This considered both prospective and retrospective reviews including randomised-controlled trials and case–control series. This study was performed following the Preferred Reporting Items for Systematic Reviews and Meta-Analyses (PRISMA) guidelines.

### Search strategy

After registration with the International Prospective Register of Systematic Reviews (Prospero CRD42022371460), we identified relevant published studies for review. Initially the search was performed in September 2022 with a final search performed in August 2023.

We built the search strategy in Ovid Medline and replicated it for use in Ovid EMBASE, Pubmed and the Cochrane Library. We searched for English language papers with the following search terms (Appendix A):

1. Femur OR femoral
2. Fracture
3. Femoral Fracture
4. Distal OR supracondylar
5. Fracture fixation OR Fracture fixation, Intramedullary OR Fracture fixation, Internal
6. Aged OR Aged, 80 and over OR Geriatric* OR elderly
7. Treatment outcome OR Patient outcome assessment OR Outcome assessment, Health care OR Outcome* OR Patient reported outcome measures OR Morbidity OR Hospital Mortality OR Mortality
8. 1 AND 2
9. 3 AND 4 AND 5 AND 6 AND 7 AND 8

These were used to search electronic databases (Embase, Medline, Pubmed, the Cochrane Library) as well as reference searches of the retrieved articles. The articles identified by the searches had duplicates removed. They were first screened for inclusion based on title and abstract and then by review of the full text article. Reference lists were searched for other potentially eligible articles manually. The search results were assessed independently by two authors (BW and TS) based on the inclusion and exclusion criteria (Table 1); disagreements were resolved by a third author (PS). Selection criteria were chosen to reflect current practice in managing these injuries and to exclude studies that included a large proportion of non-geriatric patients.

**Table 1:** Inclusion and exclusion criteria

Inclusion	Exclusion
Meta-analysis, systematic reviews, randomised controlled trials, observational studies (cohort and case-controlled), case series	Case studies, biomechanical studies, novel surgical techniques, book chapters
AO-OTA Type 33-A, -B, -C fractures	No explicitly stated postoperative weightbearing status
Lewis and Rorabeck Type I and II periprosthetic fractures	Inclusion of other fracture types in pooled data
Surgical management with lateral locked plate, retrograde intramedullary nail, or a nail-plate combination	Surgical management with a blade plate, dynamic condylar screw, or external fixation
Average patient age ≥ 60 years	Non-surgical management

### Quality assessment

Quality of the papers was assessed using the Methodological Index for Non-randomised Studies (MINORS) checklist. This tool is validated for use with non-randomised studies and produces a maximum score of sixteen for non-comparative studies and twenty-four for comparative studies. The raw scores were then converted to a percentage of maximum score which were used for the quality effects model employed in our meta-analysis.

### Data collection

We were able to extract data pertaining to patient numbers, mean age, sex distribution, type of fixation, weightbearing, complications, length of follow-up, mortality, and various functional scores. In order to divide the patients into an EWB and DWB group we used the method described by Consigliere et al. [2]. The EWB group comprised of patients that were allowed to fully weight bear (FWB), weight bear as tolerated (WBAT) and partial weight bear > 50% (PWB). The DWB group included patients that were non-weightbearing (NWB), touch or toe-touch weightbearing (TWB) postoperatively. Study data (Table 2) and quantitative data (Table 3) were collected for subsequent analysis.

**Table 2:** Included studies (LLP – Lateral locked late, NPC – Nail-plate combination, RIMN – Retrograde Intramedullary nail, EWB – Early weightbearing, DWB – Delayed weightbearing)

Study	Study Design	EWB/NWB	Follow up	Mean age (range)	Patients (% Female)	Fracture Types	Fixation Type
Giddie et al. (2015)	Retrospective Case-series	EWB	123 days (mean)	80.6 (51–103)	56 (94.6%)	Both	RIMN
Smith et al. (2016)	Retrospective Case-series	EWB	12 months	74 (52–89)	54 (68.5%)	Periprosthetic	LLP
Hussain et al. (2018)	Retrospective Case-series	EWB	11.4 months	82 (68–90)	9	Periprosthetic	NPC
Consigliere et al. (2019)	Retrospective Comparative	EWB	3 months	63.7 (44–80)	13 (100%)	Native (33A/B/C)	LLP
Kanakaris et al. (2019)	Prospective RCT	EWB	9 months	77	40 (85%)	Both	LLP
Liporace et al. (2019)	Retrospective Case-series	EWB	12.5–26 weeks	74.8	15 (60%)	Both	NPC
Bruggers et al. (2020)	Retrospective Comparative	EWB	12 months	74	11 (64%)	Both	LLP
Keenan et al. (2021)	Retrospective Comparative	EWB	1.0–9.1 years	83.1	28 (85.7%)	Periprosthetic	LLP
Lieder et al. (2021)	Retrospective Comparative	EWB	6 months	75.5	56	Both	LLP/RIMN
Paulsson et al. (2021)	Prospective RCT	EWB	52 weeks	79.2	11 (90.9%)	Both	LLP
Striano et al. (2022)	Retrospective Comparative	EWB	180 days (minimum)	82	21	Both	LLP
Ebraheim et al. (2012)	Retrospective Case-series	DWB	7.6 months	75	27	Periprosthetic	LLP
Hoffman et al. (2012)	Retrospective Case-series	DWB	22 months	73.2 (54–95)	31 (86.1%)	Periprosthetic	LLP
Khursheed et al. (2015)	Prospective Case-series	DWB	12 months	66.5 (60–70)	25 (76%)	Native (33A)	LLP
Christ et al. (2018)	Retrospective Case-series	DWB	12–108 weeks	73.9 (17–95)	40 (82.5%)	Periprosthetic	LLP
Consigliere et al. (2019)	Retrospective Comparative	DWB	3 months	61.1 (40–82)	32 (59%)	Native (33A/B/C)	LLP
Bruggers et al. (2020)	Retrospective Comparative	DWB	12 months	75	35 (74.3%)	Both	LLP
Keenan et al. (2021)	Retrospective Comparative	DWB	1.0–9.1 years	76.8	13 (86.7%)	Periprosthetic	LLP
Lieder et al. (2021)	Retrospective Comparative	DWB	6 months	76.4	79	Both	LLP/RIMN
Nam et al. (2022)	Retrospective Comparative	DWB	12–26 months	77 (64–87)	82 (59.7%)	Native	LLP
Paulsson et al. (2021)	Prospective RCT	DWB	52 weeks	81.3	18 (83.3%)	Both	LLP
Striano et al. (2022)	Retrospective Comparative	DWB	180 days (minimum)	68.2	144	Both	LLP

**Table 3:** Recorded outcomes of included studies (EWB—Early weightbearing; DWB—Delayed weightbearing)

Study	Weightbearing	Patients	Non-union	Malunion	Infection	DVT/PE	Mortality
Consigliere et al. (2019)	EWB	13	0	0	0	0	-
	DWB	32	0	4	0	0	-
Christ et al. (2018)	DWB	40	2	0	4	1	2
Hoffman et al. (2012)	DWB	36	8	5	4	1	-
Khursheed et al. (2015)	DWB	25	0	0	2	0	-
Liporace et al. (2019)	EWB	15	0	0	1	0	1
Lieder et al. (2021)	EWB	56	5	8	1	0	-
	DWB	79	8	13	4	0	-
Smith et al. (2016)	EWB	54	1	2	1	10	2
Ebraheim et al. (2012)	DWB	27	1	0	0	0	-
Hussain et al. (2018)	EWB	9	0	0	0	1	0
Paulsson et al. (2021)	EWB	11	0	0	0	0	1
	DWB	18	0	0	1	1	3
Bruggers et al. (2020)	EWB	10	0	0	0	0	0
	DWB	33	0	0	0	0	3
Striano et al. (2022)	EWB	21	1	1	1	0	6
	DWB	144	17	3	10	0	13
Giddie et al. (2015)	EWB	56	1	0	4	1	16
Kanakaris et al. (2019)	EWB	40	5	2	0	0	4
Nam et al. (2022)	DWB	82	2	0	10	0	-
Keenan et al. (2021)	EWB	28	2	3	1	0	4
	DWB	15	2	2	2	1	2

### Data synthesis

A quality effects Meta-analysis was possible to calculate pooled prevalence rates for non-union, malunion, infection and mortality as they relate to weightbearing. This was chosen over a random-effects model as it uses the quality of the study in determining the inverse variance. In this way, the weights allocated to individual studies were based on their quality rather than randomly allocated. In addition to this, we grouped studies according to quality as of poor, moderate or good quality. We then ran the analysis, again using a quality effects model, once with only high-quality studies, and once excluding only low-quality studies. The results of these analyses also showed no significant difference between the weightbearing and non-weightbearing groups. Sensitivity analyses were also performed to compare outcomes for LLP, RIMN and NPC by pooling studies that included only a single fixation type. All calculations were performed using Microsoft Excel and the MetaXL extension (version 5.3). Functional data were not appropriate for meta-analysis.

## Results

**Fig. 1:**
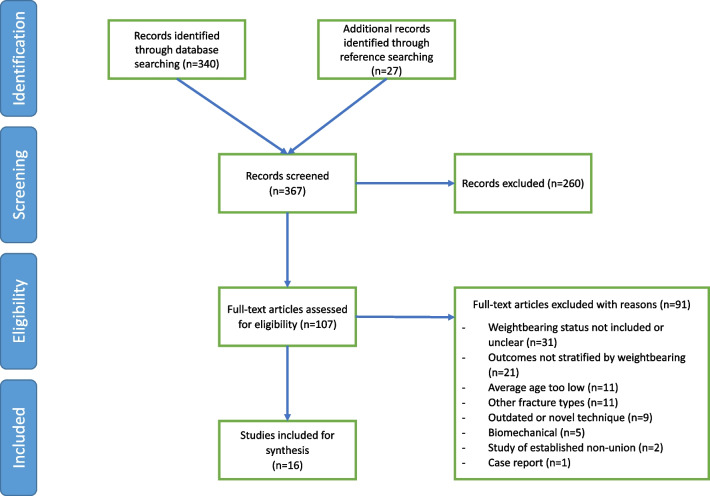
PRISMA flow diagram

The search strategy identified 340 potential articles and a search of references yielded 27 additional articles. These articles were screened for inclusion based on the inclusion/exclusion criteria by two of the authors. One hundred and seven articles were included as potentially eligible after screening of titles and abstracts and sixteen were subsequently included for analysis (Fig. 1).

A total of 844 patients were included across the sixteen studies, 531 in the DWB group and 313 in the EWB group. In the EWB group, 58.5% were treated with LLP, 33.9% were treated with RIMN and the remaining 7.7% were treated with a nail-plate combination (NPC). In the DWB group 93.8% were treated with LLP and 6.2% were treated with RIMN.

Five of the included studies allowed early postoperative weightbearing [17–21], five studies managed patients with delayed weightbearing [22–26] and six studies had an EWB and a DWB treatment group [2, 27–31].

The quality assessment graded most papers as of moderate or poor quality (Table 4).

**Table 4:** Quality assessment

Study	Case-Series/Comparative	MINORS Score	Quality Score
Consigliere et al.	Comparative	15/24	0.625
Christ et al.	Case-series	7/16	0.438
Hoffmann et al.	Case-series	9/16	0.563
Khursheed et al.	Case-series	11/16	0.688
Liporace et al.	Case-series	7/16	0.438
Lieder et al.	Comparative	16/24	0.667
Smith et al.	Case-series	10/16	0.625
Ebraheim et al.	Case-series	6/16	0.375
Hussain et al.	Case-series	8/16	0.500
Paulsson et al.	Prospective RCT	22/24	0.917
Bruggers et al.	Comparative	17/24	0.708
Striano et al.	Comparative	17/24	0.708
Giddie et al.	Case-series	11/16	0.690
Kanakaris et al.	Prospective RCT	23/24	0.958
Nam et al.	Case–control	11/16	0.688
Keenan et al.	Comparative	18/24	0.750

Overall, the heterogeneity between studies prevented statistical pooling for functional outcomes but the patient demographics and data available allowed a meta-analysis of non-union, malunion, infection rates, thromboembolic events, and mortality using a quality effects model and the Chi-squared test.

### Non-union

**Fig. 2:**
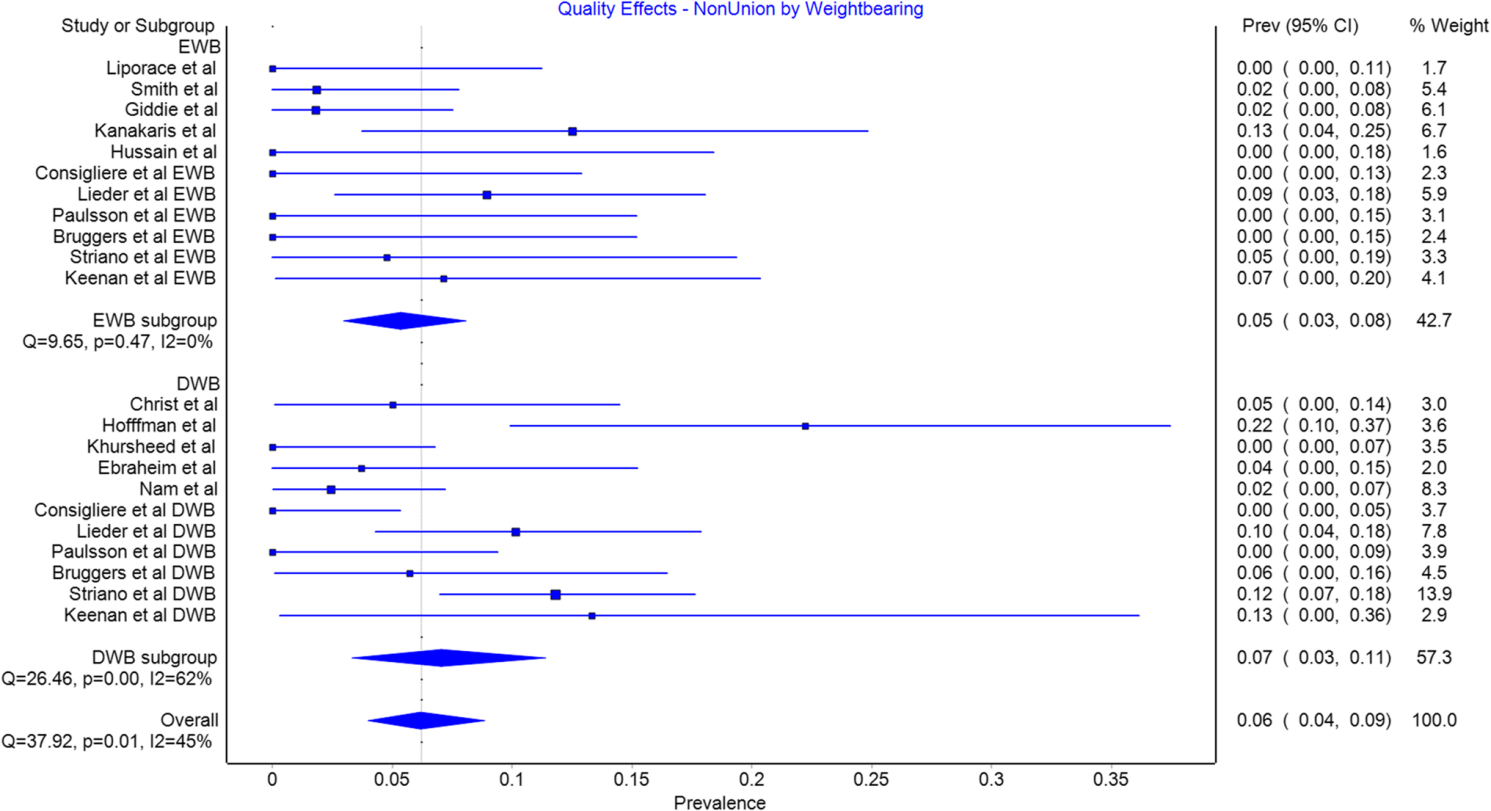
Non-union by weightbearing

All studies included non-union rates in their analysis. There was a total of 42 non-unions in the DWB group with a prevalence of 0.07 (CI 0.03 to 0.11) and 15 in the EWB group with a prevalence of 0.05 (CI 0.03 to 0.08). This difference was not significant (Fig. 2).

### Malunion

**Fig. 3:**
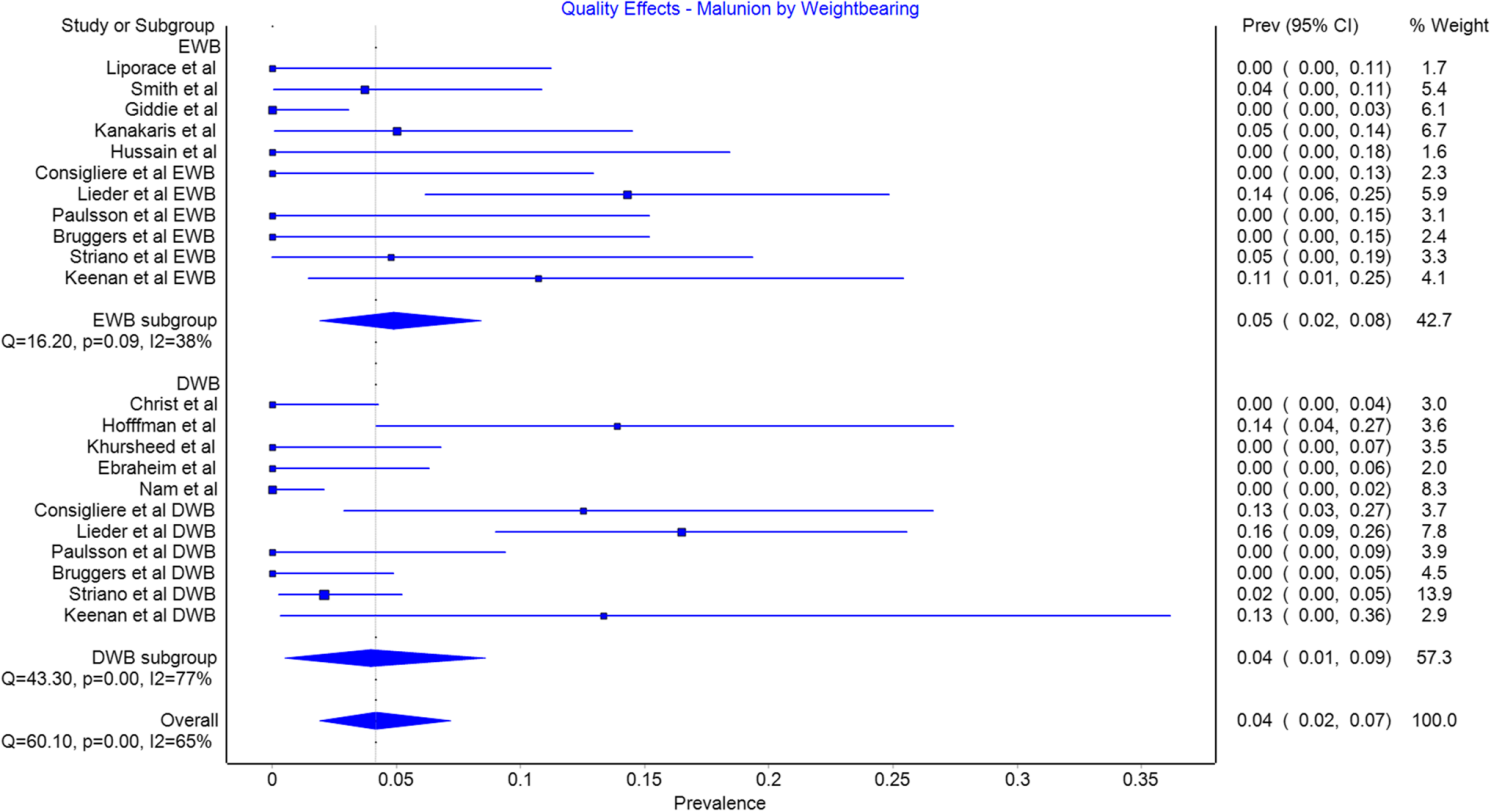
Malunion by weightbearing

Malunion was an included outcome in all studies, The prevalence was 0.05 (CI 0.02 to 0.08) in the EWB group and 0.04 (CI 0.01 to 0.09) in the DWB group (Fig. 3). There was no significant difference in prevalence between groups with 16 in the EWB group and 27 in the DWB group.

### Infection

**Fig. 4:**
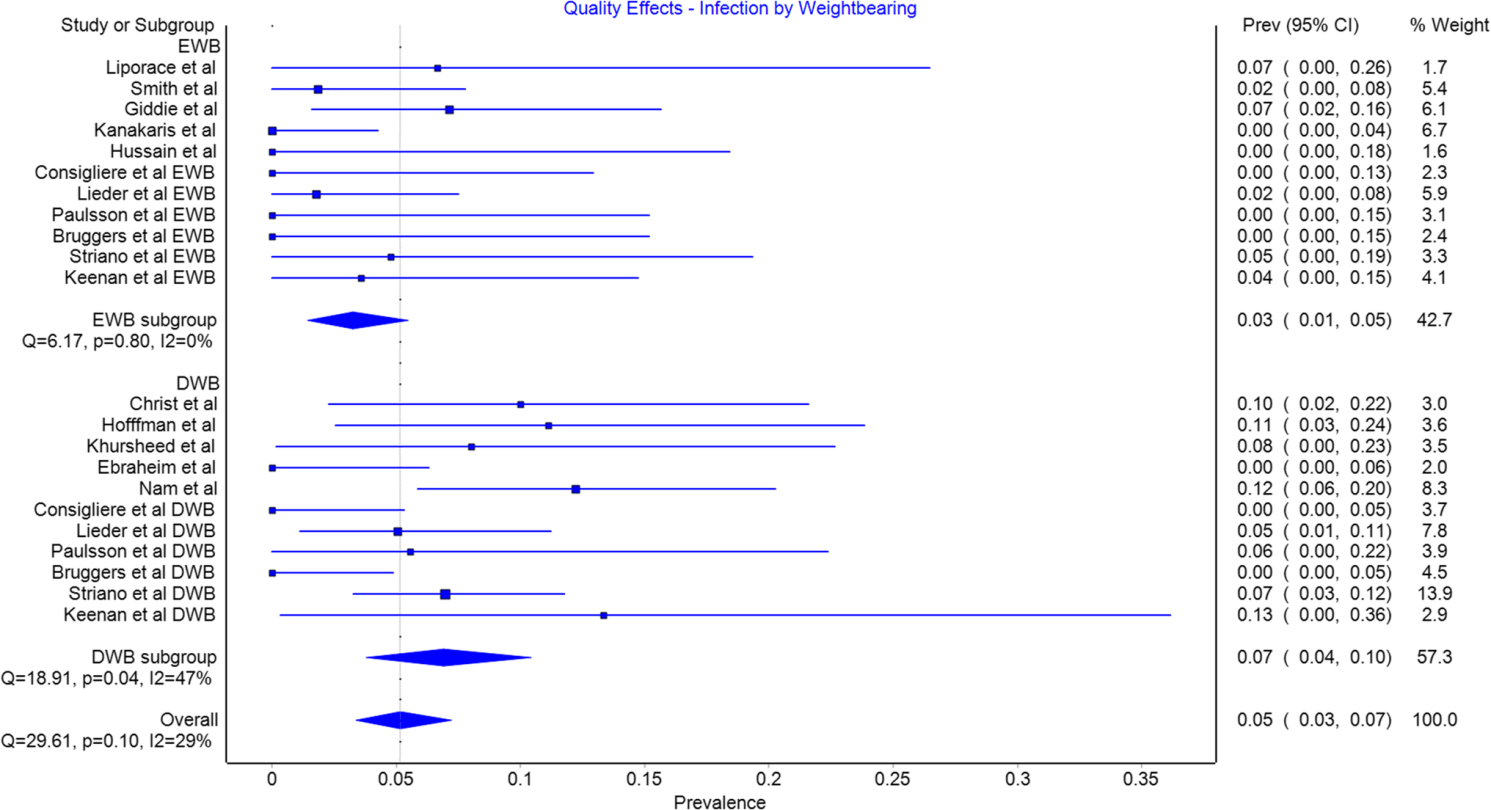
Infection by weightbearing

Infection was included as an outcome in all studies, 9 patients in the EWB group and 37 patients in the DWB group had infections but this difference was not significant (Fig. 4). The prevalence in the EWB group was 0.03 (CI 0.01 to 0.05) and 0.07 (0.04 to 0.10) in the DWB group.

### Mortality

**Fig. 5:**
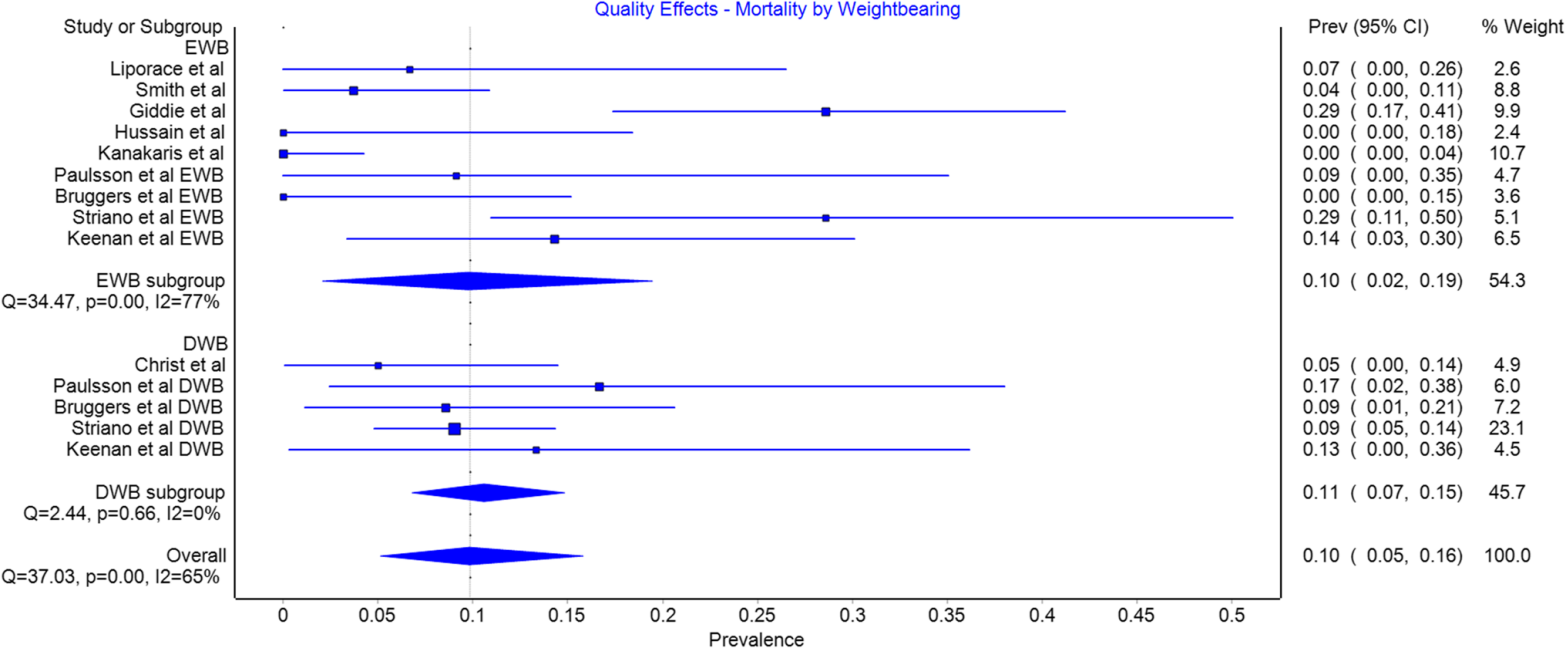
Mortality by weightbearing

Nine of the studies reported mortality [17–22, 28–31] with a prevalence of 0.10 (CI 0.02 to 0.19) in the EWB group and 0.11 (CI 0.07 to 0.15) in the DWB group. This difference was not statistically significant (Fig. 5).

### Thromboembolism

**Fig. 6:**
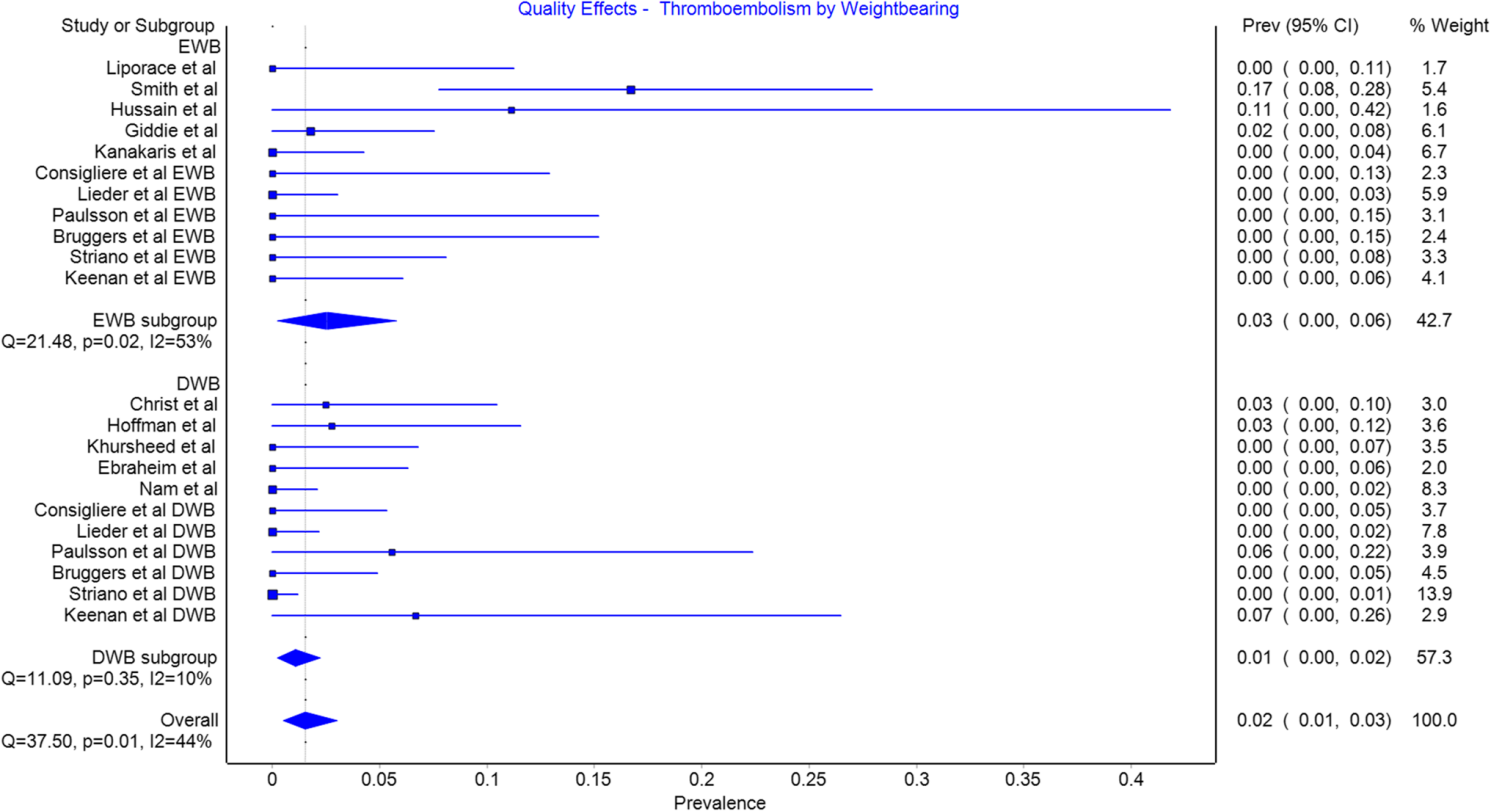
Thromboembolism by weightbearing

Deep venous thrombosis and pulmonary embolism were included as complications in all studies. There were 11 events in the EWB group with a prevalence of 0.03 (CI 0.00 to 0.06) and 4 events in the DWB group with a prevalence of 0.01 (CI 0.00 to 0.02) (Fig. 6). This result was not significant and 9/11 events in the EWB group arising from a single study. Repeated analysis excluding this study also demonstrated no significant difference between EWB and DWB.

### Sensitivity analysis

There were no significant differences between the weightbearing and non-weightbearing groups when grouping by study quality. Furthermore, repeated analysis of outcomes by fixation type (LLP, RIMN, NPC) did not show any difference between implants.

### Functional outcomes

Five studies included functional outcome scores and an additional five included mobility after injury. Unfortunately, a lack of consistency in outcome measures used made them unsuitable for meta-analysis.

Two of studies that reported mobility after injury were in the DWB group [22, 23], two were from the EWB group [17, 20], and one had both an EWB and a DWB group. The two studies in the DWB group did not report pre-injury mobility. The study by Keenan et al. [31] included pre-injury mobility as well as post-injury mobility for both EWB and DWB groups but there was no comparison of mobility status for individual patients. In addition, post-operative mobility was unavailable for 32% of patients in the EWB group and for 20% of patients in the DWB group (Table 5).

**Table 5:** Mobility after injury (EWB – Early weightbearing; DWB – delayed weightbearing)

Study	Weightbearing Status	Patients	Return to preinjury mobility (%)	Mobilise with aids (%)	Mobilise without aids (%)	Non-ambulant (%)
Christ et al	DWB	28	-	13 (46%)	15 (54%)	0 (0%)
Hoffman et al	DWB	35	-	23 (66%)	12 (34%)	0 (0%)
Liporace et al	EWB	15	11 (73%)	9 (60%)*	5 (33%)	0 (0%)
Smith et al	EWB	52	38 (73%)	-	-	-
Hussain et al	EWB	9	5 (56%)	5 (56%)Ɨ	3 (33%)	1 (11%)
Keenan et al	EWB DWB	28 15	- -	7 (25%) 7 (47%)	8 (29%) 3 (20%)	4 (14%) 2 (13%)

*Six patients required aids to mobilise prior to injury

ƗThree patients required aids to mobilise prior to injury

Two studies included EuroQol 5-dimension (EQ-5D) instrument scores but only one stratified scores by weightbearing status [28].

In the study by Paulsson et al., additional outcome measures were also used and stratified by weightbearing status including short musculoskeletal functional assessment (SMFA), visual analog scale (VAS) for pain, and timed up-and-go (TUG) tests. The TUG test was five seconds faster in the FWB than in the PWB group, but this was not statistically significant. They did not show any statistically significant differences in SMFA, EQ-5D, or VAS scores between groups.

## Discussion

Geriatric lower limb fractures are often life-altering events for the affected population. DFF are associated with significant morbidity, mortality, and loss of independence [5–7, 32–34]. The demographics and outcomes of DFF mirror those of geriatric proximal femoral fractures [1, 29, 33] where the standard of care has shifted to multidisciplinary care, early operative intervention and immediate weightbearing with early mobilisation [35, 36]. These injuries place a burden on the healthcare system with loss of independence, long hospital stays and management of complications.

There is a well-established link between mobility as measured by the Parker mobility score, 1 year mortality, discharge disposition and dependency in geriatric patients [37–39], highlighting the importance of postoperative mobility. Prolonged immobility in geriatric patients exacerbates pre-existing sarcopenia and loss of functional skeletal muscle in these patients can be irreversible [13, 14], supporting the importance of minimising immobility in patients with DFF. In geriatric hip fracture patients, weightbearing is an all-or-nothing proposition [11] and any form of weightbearing restriction increases their immobility, as well as related complications and risk of falls [9, 40]. With their similar demographics, geriatric DFF should be treated with the same weightbearing principles applied to hip fracture patients. This is further supported by our study; for the studies that included post-operative mobility as an outcome, 29% of patients were unable to return to pre-injury mobility and only 41% of patients were able to mobilise without aids after their injury.

Our meta-analysis supports the safety of EWB with no significant difference in complications, but it is limited by the heterogeneity and relatively poor quality of the included studies. Robust evidence, in the form of a large prospective randomised trial, to support either the safety of early weightbearing or the long-term benefit of early weightbearing is lacking. One study [28] randomised geriatric femoral fractures to either FWB or 30% PWB but found no statistically significant difference in functional outcomes or patient length-of-stay (LOS). This may be due to small sample sizes in each group resulting in an underpowered study. Although LOS was equivalent between the groups, all the patients (11/11) in the FWB group were discharged to their homes while 3/18 in the PWB group were discharged to nursing homes.

No high level evidence exists for any aspect of management for DFF, including choice of implant; this limits the ability of surgeons to use literature to guide best practice [41]. Literature around DFF is primarily retrospective, with a focus on case series comparing outcomes with various implants, the biomechanical feasibility of weightbearing or implant characteristics that may predispose to failure after surgical management. Biomechanical studies have shown that both lateral locked plating and retrograde intramedullary nailing have their own strengths and weaknesses as a construct [42] and suggest that lateral locked plating is insufficient for EWB which may be better facilitated with retrograde intramedullary nails [43, 44]. The hesitation from surgeons when it comes to EWB may be based on these biomechanical studies as well as published studies suggesting failure rates between 9–20% after lateral locked plating after DFF [45–48]. While our subgroup analysis did not show any difference between constructs when compared by weightbearing status, the sample size for both RIMN and NPC was too small to allow us to draw any broad conclusions in this regard. Few studies have suggested criteria to allow EWB and those that have are largely based on expert opinion rather than evidence [3, 49]. Some authors have advocated novel fixation to deal with perceived deficiencies in the commonly used implants [17, 50, 51] but these can be technically challenging as well as costly when it comes to combining implants.

In the more recent literature, there has been increased advocacy for EWB after geriatric DFF. This has been partly encouraged by the improved outcomes for proximal femoral fractures treated according to the NICE guidelines but also as a response to biomechanical studies that suggest that weightbearing may result in decreased non-unions by increasing longitudinal motion at the fracture site and stimulating callus [10, 52]. This is reflected by the relative recency of the studies that included EWB included in our analysis. Novel fixation techniques may also play an eventual role with multiple papers demonstrating excellent outcomes with nail-plate fixation and immediate weight-bearing as tolerated [17, 20, 53].

In addition to the papers included in our systematic review, a retrospective study of 122 patients with 127 fractures was performed by Poole et al., in 2017. In this study, 107 of 127 patients were allowed EWB postoperatively with good results. The study was excluded from analysis as the outcomes were not stratified based on weightbearing status but their overall results demonstrated union rates of 95% in patients with complete sets of radiographs (81/85) and only 3 of the 127 fractures required reoperation for loss of fixation prior to union [54].

Most of the studies included in this review had a high risk of bias due to their retrospective nature and due to the nature of the involved patient populations. The results of the meta-analysis demonstrate non-inferiority of outcomes with EWB compared to DWB with no significant difference in non-union, malunion, infection, thromboembolic events, or mortality.

Limitations of this study are largely due to the largely retrospective nature of the included studies, which reflects the literature related to DFF in general. In addition, the inclusion of studies with an average age of ≥ 60 years means that in at least two of the studies, patients were included in the analysis that are not a part of the population of interest. However, the effect of these few patients on the overall results is likely to be minimal.

Heterogeneity of the studies also presented a major barrier to meta-analysis of functional outcomes, including pain, mobility, requirements for mobility aids and knee range of motion. Obtaining functional outcome scores is understandably difficult or impossible with retrospective studies but even between prospective studies there is a lack of consistency with the scoring systems used.

There are significant barriers to both the adoption of both multidisciplinary management and to early unrestricted weightbearing for patients with distal femoral fragility fractures. Multidisciplinary management typically involves either an orthogeriatric or hospitalist service to aid surgeons in optimising these medically complex patients for theatre. Geriatric proximal femur fractures already represent a significant workload for these physicians and a push to include DFF under their umbrella of care needs to be supported by evidence of improved outcomes. To do so would require increases in funding to these areas which would need to be offset by either an expected reduction in complications, length-of-stay or return to prior levels of independence. Prospective studies to support these changes are currently lacking and should be a focus of future studies to afford these patients the same care afforded to patients with proximal femoral fragility fractures.

## Supplementary Information

Below is the link to the electronic supplementary material.

Supplementary file1 (DOC 74 KB)

## Data Availability

No datasets were generated or analysed during the current study.
